# Differential achievements in childhood immunization across geographical regions of Pakistan: analysis of wealth-related inequality

**DOI:** 10.1186/s12939-018-0837-6

**Published:** 2018-08-17

**Authors:** Owais Raza, Fahad Saqib Lodhi, Esmaeil Khedmati Morasae, Reza Majdzadeh

**Affiliations:** 1 Department of Epidemiology and Biostatistics, School of Public Health, International campus, Tehran University of Medical Sciences, 5th Floor, Poursina Street, Keshavarz Boulevard, PO Box: 14155-6446, Tehran, Iran, Islamic Republic of; 2 Centre for Systems Studies, Hull University Business School, Hull University, Hull, HU6 7RX UK; 3 Knowledge Utilization Research Center and Center for Community-Based Participatory-Research, Tehran, Iran

**Keywords:** Inequality, Childhood immunization, Achievement index, Pakistan demographic and health surveys

## Abstract

**Background:**

Childhood immunization is one of the most cost-effective interventions for child health. Still, many children are not able to receive completed immunization status. Wealth – related inequality in immunization is considered a major reason for equitable coverage of immunization in Pakistan. Therefore, we examine wealth-related inequality in completed childhood immunization and to assess achievement indices across geographical regions in Pakistan.

**Methods:**

The analysis was based on a nationally representative demographic and health survey (DHS) of Pakistan, conducted in 2012–13. We examined completed childhood (12–23 months) immunization in the various regions of the country and we used concentration, extended concentration and achievement indices to demonstrate inequality across geographical regions in Pakistan.

**Results:**

Inequality in completed childhood immunization was seen in Pakistan with concentration index (CI) of 0.181 (95% CI: 0.164–0.209). Regions with high average of complete immunization showed lower inequality except for Sindh. Despite having better average immunization coverage in Kyber Pakhtunkhwa, the relative change of 128% in concentration index (CI) from C2 (standard CI) to C5 (when poorer quantile received highest weights) shows this to be also the most inequitable regions. Four parameters of inequality aversion (v = 2, 3, 4 & 5) demonstrated that ‘dis – achievement’ in completed immunization is densely concentrated among the poorer regions. Balochistan, Sindh and Gilgit Baltistan exhibited broader inequality gaps (93.75%, 83.35%, and 54.93%, respectively) at higher aversion parameter.

**Conclusions:**

As hypothesized, achievement index uncovers ‘penalized’ immunization coverage amongst the poorest population. Thus any policy that stringently focuses on improving average immunization rate without any strategy to deal with inequality will only improve immunization rate within wealthier groups. Based on these results, it is advisable to public health policy makers to use both aspect of information: average and degree of inequality in immunization coverage.

**Electronic supplementary material:**

The online version of this article (10.1186/s12939-018-0837-6) contains supplementary material, which is available to authorized users.

## Background

Immunization is one of the safest and strongest interventions for preventing childhood diseases and worldwide preventing nearly 3 million deaths per year. Despite this success, unfortunately, nearly 20 million infants miss at least one dose of immunization [1]. WHO reported in their ‘State of Inequality: Childhood Immunization’ report, that out of 69 countries, one quarter have a national average of less than 50% [2]. Secondary analysis of Demographic and Health Survey (DHS) and Multiple Indicator Cluster Surveys (MICS) from 54 low- and middle-income countries has shown that in terms of childhood immunization, highly inequitable countries are from North African region, followed by Madagascar, India and Pakistan [3]. Many countries have shown progress in achieving MDG’s target while some countries, like Pakistan, continue to struggle towards meaningful improvement in average immunization rate. Pakistan suffers significant amount of inequality, with the difference up to 60% points between poorest and wealthiest strata of population [2]. Published literature in Pakistan indicates marked inequality in childhood immunization coverage at provisional and district levels. For instance, the national coverage of DPT3 was 80% as reported by WHO/UNICEF in World Health Statistics 2013 reporting on 2011 data [4], which masks unequal immunization coverage between Baluchistan (49%) and Punjab (86%) in that year [5].

In the field of public health, classical approaches for measuring inequalities are either quintile-based or regression analyses. The major limitation with these analytical methods is that they quantify the amount of inequality by comparing two wealth strata. Moreover, within stratum the population is assumed to be affected similarly by the inequality. In order to quantify the degree of wealth-related inequality in outcome, concentration index (CI) has been extensively used [6–9]. This index quantifies the degree to which health services, for example are better targeted towards poorer or wealthier segment of the population [10]. Alternatively, extended concentration index allows us to modify the weight attached to the health share. By doing so, one can observe how inequality changes as the attribute to inequality changes [10].

In this paper, we intend to provide evidence that measures of inequality when combined with information on the average immunization coverage can reveal the burden of wealth-related inequality among poorer socioeconomic group, especially in a context when improving average coverage may not necessarily improve immunization coverage of the less well-off stratum. To achieve this, our study has two objectives; (a) to quantify wealth-related inequality in completed childhood immunization in Pakistan, and (b) to adjust the average coverage of childhood immunization with accompanying data to show inequality across the geographical regions of Pakistan.

## Methods

### Data

The dataset used to estimate wealth – related inequality in childhood immunization was the Pakistan Demographic and Health Survey (PDHS) 2012–13 [11], which was a nationally representative household survey. The PDHS 2012–13 was the third survey conducted as a part of the MEASURE DHS program international series. In PDHS 2012–13, a two – staged sampling design, stratified on region and place of residence (urban/rural) was adopted to provide reliable estimates at national, provisional and urban and rural levels. All urban areas were divided into smaller areas, known as enumeration block. Each block consisted of 200–250 households on average. In rural areas, the list of villages developed through the 1998 population census was used. Within each enumeration block, listing of household was conducted that served as the sampling frame for the selection of household in the second stage. In the second stage of sampling, a fixed numbers of households (i.e. 28 households) were selected through systematic sampling technique with a random start. The sample universe consists of all four provinces and Gilgit-Baltistan and Islamabad Capital Territory (ICT) (for further details on sampling methods, see appendix B of Pakistan Demographic and Health Survey 2012–13 [11]). This design made the use of weighted analysis (weights provided within PDHS dataset) mandatory, and adjusted for cluster sampling frame with cluster as the primary sampling unit and household as the secondary sampling unit. With the response rate of 93.06%, 13,558 ‘ever – married’ women aged 15–49 years were interviewed and information regarding childhood’s immunization status was gathered for 2074 children aged between 12 and 23 months [11].

The dependent variable for this study was immunization status of children aged 12–23 months (‘1’ for complete immunization and ‘0’ otherwise). To define completed childhood immunization, we used WHO’s definition of complete immunization is defined as; a child aged 12 to 23 months who, at the time of survey, had received the following vaccines: one dose of the vaccine against tuberculosis; three doses of the vaccine against diphtheria, pertussis, and tetanus; three doses of polio vaccine (excluding polio vaccine given at birth); three doses of the vaccine against hepatitis B; three doses of the vaccine against Haemophilus influenza type b; and one dose of measles vaccine.

The wealth index provided within PDHS data set was generated by applying principal component analysis on household assets that was used as a proxy. This wealth index is based on household ownership of consumer goods (such as radio, television, whitegoods); dwelling characteristics; type of drinking water source; toilet facilities and other characteristics related to the household’s socio-economic status. Such wealth index serves as an indicator of household wealth that has been consistent with household income in various studies [12, 13].

### Methodology

Inequality has been comprehensively describe by the rate ratios of immunization coverage among wealthiest and poorest quintile groups [14–16] but it fails to reflect the experience of the entire population at risk. We therefore used concentration index (CI) that overcomes the limitation of rate ratios using quintiles. In addition, we used an index of “achievement” proposed by Wagstaff which adjusts progress in mean coverage, depending on the accompanying change in inequality [17]. All of the statistical analyses were completed in STATA v13 [18].

### Wealth – related inequality

Wealth – related inequalities in health have been measured in various ways [19–21]. For this study we adopted the Wagstaff [22] concentration index (CI) method which measures cumulative distribution of outcome variable (i.e. completed childhood immunization) ranked by wealth indicator as a deviation from equal distribution. The sign of the CI represents the direction of relationship between completed childhood immunization and position in the distribution of wealth index across the sample. The magnitude of CI reflects both the strength of the relationship and the degree of variability in completed childhood immunization. See Additional file 1 for the formula for the concentration index. We are reporting this index with concentration curve, which plots cumulative proportion of complete immunization in children 12–23 months (on y – axis) against cumulative proportion of children, ranked by their household’s wealth (on x – axis). In a population where each individual share equal amount of health outcome (i.e. completed childhood immunization) the concentration curve will be a 45-degree line. This is known as the line of equality. On the other hand, if the health outcome takes lower values among poorer segment of the population, then the concentration curve will lie below the line of equality.

### Extended concentration index

Despite strength of concentration index measure, it implicitly carries a fixed view of where in the wealth distribution reduction in inequality will matter most. Whereas, extended concentration index allows attitudes of inequality to be made explicit and this provides us a liberty to examine other values of inequality aversion parameter (*v* in eq. 2 of Additional file 1).

When v = 1, health of each subject is weighted equally and in this case value judgement is that wealth-related inequality does not matter in the distribution of health outcome. Therefore, CI = 0 when v = 1, no matter how unequally health outcome is distributed across the wealth index. Now, as we raise the v above 1, weights attached to the health of poor person rises, while weight for the health of rich person decreases. When inequality aversion parameter v = 2 [i.e. C(2)], the weight becomes similar as in the standard concentration index. As we keep increasing the level of v, poor income group will receive more weights than the wealthier group, such that when v = 5 [i.e. C(5)], individuals within poorest wealth quintile have highest weights. To clarify further, suppose the health outcome is a health advantage (e.g. completed immunization) and is more concentrated among the rich households and also, suppose that we assign more weight on the health of poor, and the extended CI (C (*v*)) shows a increase when the degree of inequality aversion parameter (*v*) increases.

### Achievement index

To capture both the mean of the complete immunization and the degree of inequality across wealth strata, achievement index was applied [10]. It is defined as a weighted average of the health levels of the various people in the sample, in which higher weights are attached to poorer people than to wealthier people. See Additional file 1 for a definition of achievement index. In a situation where health variable is health advantage (completed immunization, for instance) and is more concentrated within wealthier population, the attached weight (1 – *C* (*v*)) will help to decrease *I* (*v*) beyond the mean, rendering the achievement worse than it would appear when solely focusing on mean. When *v* = 1 in eqs. 3 & 4 of Additional file 1, achievement index merely reflects the average complete immunization, as all individual received equal weights ignoring any wealth – related inequality. To enable achievement index to capture inequality across all economic strata, we assigned higher weight to the lowest ranked groups compared to the higher ranked groups. Therefore, higher weights are allocated for less well-off segment of the population when v > 1.

## Results

From Table 1, it is clear that proportion of completed childhood (12–23 months) immunization varies according to the wealth quintile, ranging from 23.4% within poorest stratum to 75.4% within richest wealth quintile. It is evident too that immunization coverage has a wide gap between urban and rural areas of Pakistan.

**Table 1:** Proportion of complete immunization in children (12–23 months), by region and wealth quintiles, in Pakistan, 2012–13

Characteristics	Proportion (95% CI)
Type of residence	
Urban	65.79 (62.02–69.37)
Rural	48.43 (45.85–51.03)
Wealth quintiles	
Poorest	23.44 (19.78–27.56)
Poorer	53.92 (49.26–58.52)
Middle	57.40 (52.48–62.17)
Richer	65.37 (60.78–69.69)
Richest	75.39 (70.50–79.70)

**Fig. 1:**
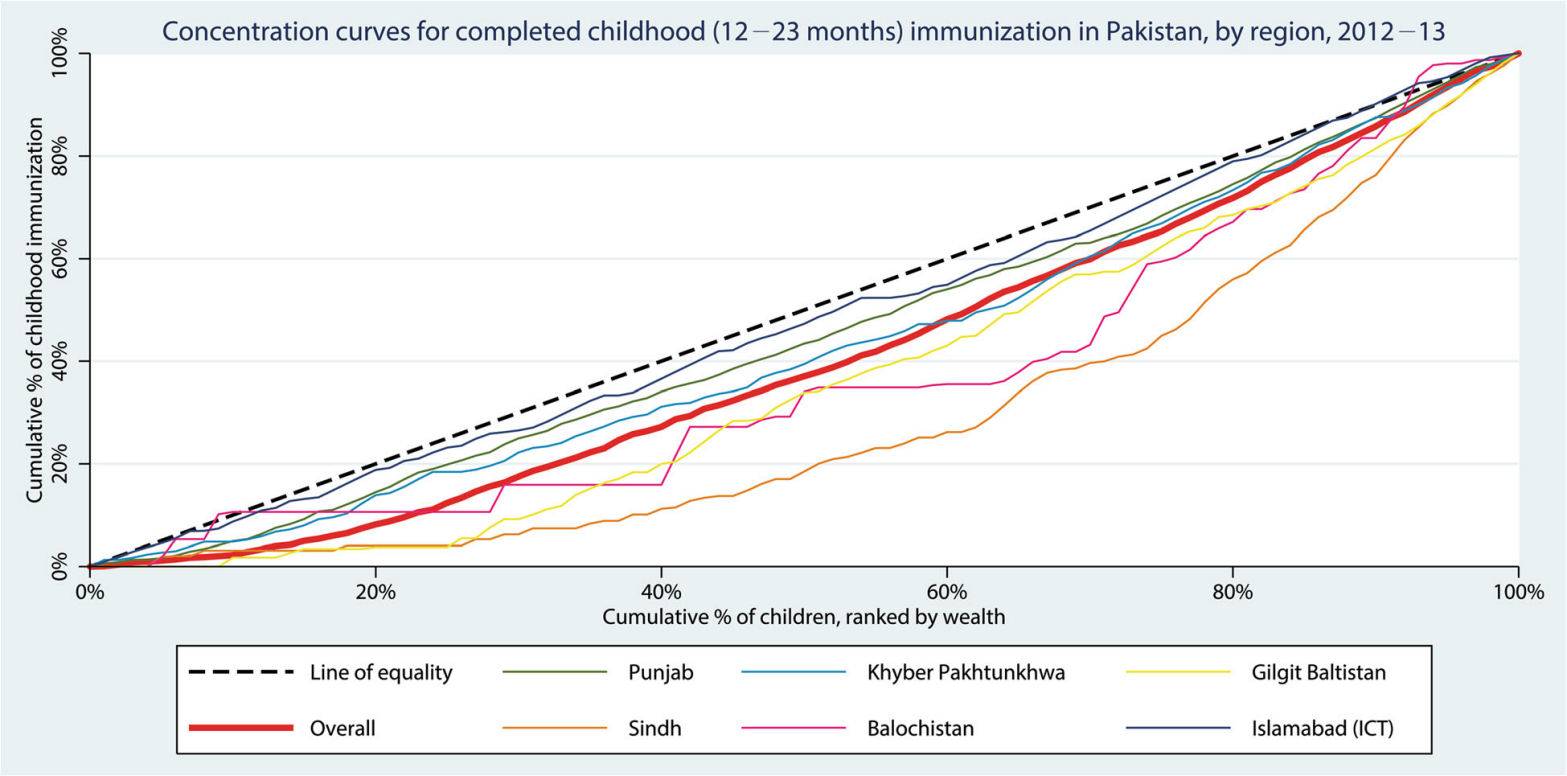
Concentration curves for completed childhood (12–23 months) immunization in Pakistan, 2012–13

The overall concentration index in our data was found to be 0.181 (95% CI: 0.164–0.209) for childhood immunization. Figure 1 shows the concentration curves for the whole population and geographical regions. Curve lies below the line of equality, interpreted as wealth – related inequality in complete immunization is in favor of children from wealthier households. In Fig. 1, concentration curves for Gilgit Baltistan, Sindh and Balochistan are under the overall concentration curve indicating that these regions face higher degree of pro-rich inequality in completed childhood immunization.

**Fig. 2:**
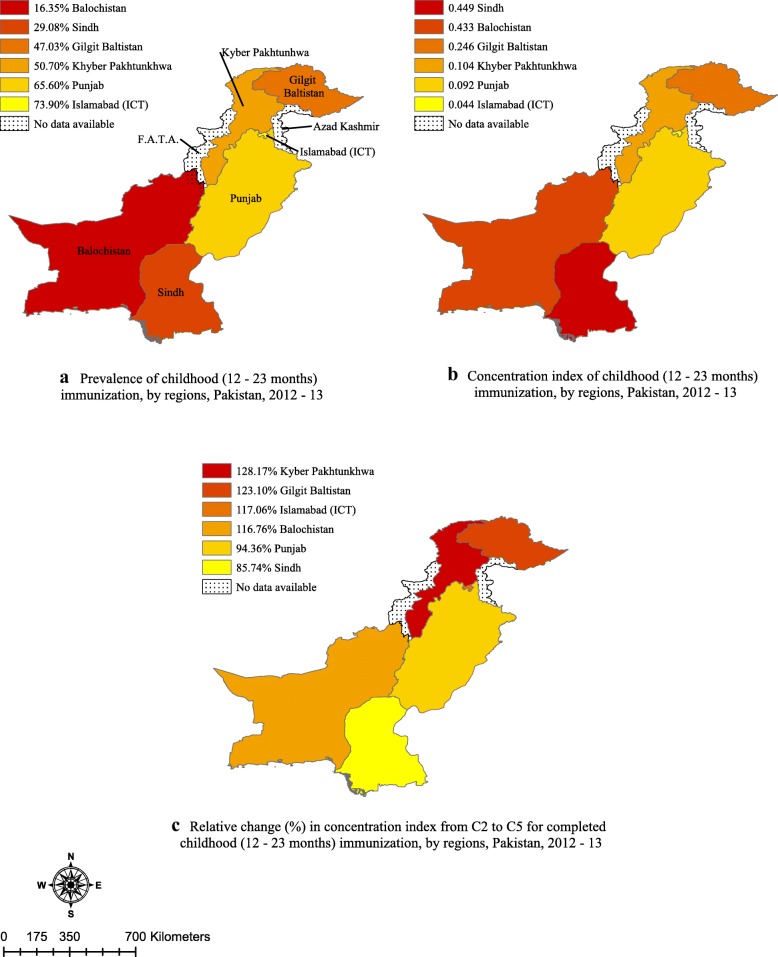
**a** Prevalence, (**b**) concentration index and (**c**) relative change in concentration index from C2 to C5 for completed childhood (12–23 months) immunization, by region, Pakistan, 2012–13

Figure 2 (a) depicts mean coverage of completed immunization along with wealth – related inequality across various regions of Pakistan. Prevalence of completed childhood immunization is less than 50% in Gilgit Baltistan, Sindh, and Balochistan. The overall concentration index (CI) for wealth – related inequality in Pakistan is thus favoring wealthier quintiles and the same pattern with varying degree of pro – rich inequality is present in all regions across the country as depicted in Fig. 2 (b). The lowest degree of wealth – related inequality is found in Islamabad (ICT), Punjab and Kyber Pakhtunkhwa (KPK). Comparing (a) and (b), we can observe remarkably that in spite of lowest prevalence of immunization in Balochistan, wealth – related inequality in completed immunization is worse in Sindh, CI = 0.45 (95% CI: 0.31–0.46) as compared to Balochistan where CI = 0.43 (95% CI: 0.01–0.45). To gain further understanding of wealth – related inequalities across various regions, we raised inequality aversion parameter (*v*) from *v* = 2 [i.e. C(2), which gives the standard CI] to *v* = 5 [i.e. C(5), when poor quantile received highest weights] and calculated ‘relative change percentage’ as depicted in Fig. 2 (c). For example, relative change of 128% in CI of inequality in KPK reflects that immunization coverage among poorest group is far lower than the other wealth quintiles. In addition to KPK, other regions such as: Gilgit Baltistan, Islamabad (ICT) and Balochistan showed more than 2-fold rise in inequality when the value of v changed from 2 to 5. This reflects the sensitivity of completed childhood immunization to wealth index. In comparison, Sindh shows a lesser degree of difference in the inequality between poorest and richer wealth quintiles with relative change of 86%.

Table 2 presents results in term of adjusted achievement scores for complete childhood immunization by geographical regions and for Pakistan. For this study, our health variable is a health advantage (completed immunization) and its distribution is skewed towards wealthier segment of the population, therefore when we raised the value of v above 1, reveals to smaller achievement of completed immunization in comparison to the average coverage when wealth-related inequality was not taken into account. For this purpose, four parameters of inequality aversion (*v* = 2 to *v* = 5) are shown, complemented with unadjusted score (*v* = 1). The justification of applying achievement index is evident from raising the value of *v* which results in decreasing immunization coverage. This indicates that ‘dis – achievement’ in complete immunization is densely concentrated among the poorer families.

**Table 2:** Inequality – adjusted achievement index scores for complete immunization in children (12–23 months), by regions, Pakistan, 2012–13

Inequality aversion^a^	*v* = 1 (mean)	*v* = 2	*v* = 3	*v* = 4	*v* = 5	Relative change from mean (%)^b^
Balochistan	0.16	0.09	0.05	0.02	0.01	93.75
Sindh	0.29	0.16	0.10	0.07	0.05	83.35
Gilgit Baltistan	0.47	0.35	0.29	0.25	0.21	54.93
Kyber Pakhtunkhwa	0.53	0.47	0.44	0.42	0.40	23.76
Punjab	0.66	0.60	0.57	0.55	0.54	17.97
Islamabad (ICT)	0.74	0.71	0.69	0.68	0.67	9.57
Overall Pakistan	0.54	0.44	0.38	0.34	0.31	42.55

^a^*v* = 1 corresponds to an equal weight on all individuals irrespective of the economic status; *v* = 2 has weight of the standard concentration index and whereas *v* = 5 gives maximum weights to individuals in poorest wealth quintiles

^b^Arranged in descending order, expect for overall Pakistan

## Discussion

This study, by using a more pragmatic methodology, is presenting distribution of wealth – related inequality in childhood immunization across geographical regions of Pakistan. Our main aim in this study was to show wealth – related inequality in completed childhood (12–23 months) immunization across various geographical regions of Pakistan, favoring wealthier households, but with various degrees among different regions. The fact that only one half of children in Pakistan are completely immunized points to poor performance in national immunization agenda. The inability to achieve MDG’s target for providing universal coverage of immunization can be attributed to geographical disparities in healthcare access. Batool S and Ahmed AM (2017) [23] found that the geographical region is one of the most influential factors in determining the inequalities in child health, including completed immunization. Using concentration index methods, recent studies have also demonstrated that Pakistan, along with India, Cameroon and Nigeria, has largest wealth-related inequality [24–26].

The methodologies employed in this analysis enabled us to look further into this matter. Region – based CI showed minimal inequality in completed immunization in Punjab and Islamabad, which is plausible as evidence from other studies have shown similar differences [23]. Extended concentration index allows using different value judgements (i.e., inequality aversion parameter) to raise weights attached to the poor and reduce it to the wealthier quintiles. Application of extended concentration index exposed varying degrees of inequality present in different socioeconomic strata (i.e. wealth quintiles). By mapping relative change in wealth – related inequality, we unveiled the unequal gap between poorest and richest quintiles across the regions. This showed that although some regions have less inequality in immunization (for example, Islamabad), they have wider gap between neighboring economical strata in comparison to region with higher inequality (for example, Sindh). Similar situation was found in India, where states have showed disparity in term of children health in general and immunization in particular [27].

As recommended by previous studies [14, 27, 28], any policy that brings improvement in the health average while ignoring its relative distribution across wealth quintile will be inaccurate and inequitable. Results from achievement index that returned inequality – adjusted percentages of completed childhood immunization, demonstrated that ‘achievement’ in immunization coverage can be ‘penalized’ for wealth – related inequality favoring the wealthier places. This phenomenon, although occurring in all the regions, is better recognized in case of KPK where regional average is almost similar to national coverage but it gets ‘penalized’ as we raised weight attached to the poorest segment of population on wealth distribution. Thus, strategies that are designed solely on average immunization coverage may continue to risk poorer segments of population. This can be observed in Balochistan and Sindh, where low immunization coverage and inequality are both present. Furthermore, when other things are kept constant, increase in health from a person to any other person from lower wealth quintile will increases the achievement index [17]. In this sense, achievement index is far more practical indicator for measuring improvement than an unadjusted parameter.

### Policy implications

This paper has brought a light on a critical issue that should be taken into account while planning an intervention for childhood immunization. In Pakistan, after the 18th constitutional amendment code that led decentralization of Ministry of Health (MoH), national health programs have been severely affected from lack of funds from international donor agencies, including national EPI [29]. Before 18th constitutional amendment on devolution of MoH, international donor agencies had one-window operation with federal MoH. Provincial and regional health ministries have never been a direct recipient of funding and therefore, international donor agencies are reluctant for providing direct funding to provincial ministries. On the other hand, immunization programs remain low priority for provincial and district governments [30]. Moreover, provincial/regional health ministries are at different levels in terms of capacity and resources [31]. Poor performance of immunization campaign by provisional government led re-launching EPI program at national level. This anomaly in devolution was seen obligatory to content international donors. Capacity building at provisional level should be undertaken to modulate influx of funds from international donors. Not all provinces are equally prepared to run immunization program without supervision of federal government. Most of the provinces, except Punjab, are not ready to scale up its capacity, for example: there is only one laboratory in Pakistan for testing efficacy of vaccines. To gain more equitable immunization rate, provisional governments should strengthen processes in terms of autonomy, planning, and monitoring of funds.

## Conclusion

Our results demonstrated that wealth – related inequality in completed immunization is inclined more towards wealthier segment of population of Pakistan. We also showed that relying solely on improving average completed immunization may be misleading and will fail to address inequality. Based on these results, it is advisable to public health policy makers to use both aspect of information; average and degree of inequality in immunization coverage to design more realistic regimen for national and sub – national childhood immunization program.

## Additional file

Additional file 1: Equations 1-4. (DOCX 24 kb)

## Abbreviations

BCG: Bacillus Calmette–Guérin
DHS: Demographic and Health Survey
DPT: Diphtheria, Pertussis, and Tetanus
EPI: Extended Program of Immunization
HepB: Hepatitis B
ICT: Islamabad Capital Territory
KPK: Khyber Pakhtunkhwa
MDGs: Millennium Development Goals
NITAG: National Immunization Technical Advisory Group
OPV: Oral Polio Vaccine
PDHS: Pakistan DHS
SDGs: Sustainable Development Goals
UNICEF: United Nations International Children’s Education Fund
WHO: World Health Organization
