# Supplementary material for: Differential achievements in childhood immunization across geographical regions of Pakistan: analysis of wealth-related inequality
Source: Int J Equity Health. 2018 Aug 17;17:122. doi: 10.1186/s12939-018-0837-6 (PMC6098575; doi:10.1186/s12939-018-0837-6)
Supplement: Supplementary file 1 — Equations 1-4. (DOCX 24 kb) [file 12939_2018_837_MOESM1_ESM.docx]

**Appendix:**

***A1: Concentration index***

For individual level data, concentration index (CI) is equal to:

$$C=1- \frac{2}{n\mu} \sum_{i=1}^{n} y_{i} \left( 1-R_{i} \right) \ldots(1)$$

where *n* is the sample size, *y_i_* is the health indicator for *i^th^* person, *μ* is the mean level of health and *R_i_* is the fractional rank of the wealth distribution of the *i^th^* person. It is noteworthy that the quantity (*y_i_ / nμ*) is the share of health (completed immunization) enjoyed by the *i^th^* person, which is the weighted summation by twice the component of the person’s fractional rank, i.e., 2(1 – *R_i_*). Therefore, the CI is simply 1 minus the sum of these weighted health shares utilized by the sample.

***A2: Extended concentration index***

The extended concentration index can be written as follows:

$$C\left( v \right)=1- \frac{v}{n\mu} \sum_{i=1}^{n} y_{i} \left( 1- R_{i} \right)^{v-1} v>1 \ldots(2)$$

where the weight attached to the health of *i^th^* person (*y_i_* / *nμ*) equals *v(* 1 – *R_i_*)^(^*^v -^* ^1)^ rather than 2(1 – *R_i_*).

***A3: Achievement index***

The achievement index can be written as follows:

$$I\left( v \right)= \frac{1}{n} \sum_{i=1}^{n} y_{i}{v \left( 1- R_{i} \right)}^{v-1} \ldots(3)$$

The equation (3) can be shown to be equal to the following:

$$I\left( v \right)= \mu\left( 1-C(v) \right) \ldots(4)$$

In equations 3 & 4, parameter *v* represents priority weight attached to the rank of the economic strata, so *I(v)* could be defined as a weighted average of complete immunization in a society where poorer people get a more attention (higher weight) compared to richer ones.
